# A member of the *Plasmodium falciparum* PHIST family binds to the erythrocyte cytoskeleton component band 4.1

**DOI:** 10.1186/1475-2875-12-160

**Published:** 2013-05-11

**Authors:** Lindsay A Parish, Deborah W Mai, Matthew L Jones, Erika L Kitson, Julian C Rayner

**Affiliations:** 1 Department of Microbiology, University of Alabama at Birmingham, 845 19th St South, Birmingham, AL, 35294-2170, USA; 2 William C Gorgas Center for Geographic Medicine, Division of Infectious Diseases, Department of Medicine, University of Alabama at Birmingham, 845 19th St. South, Birmingham, AL, 35294-2170, USA; 3 Malaria Programme, Wellcome Trust Sanger Institute, Wellcome Trust Genome Campus, Hinxton, Cambridge, CB10 1SA, UK

## Abstract

**Background:**

*Plasmodium falciparum* parasites export more than 400 proteins into the cytosol of their host erythrocytes. These exported proteins catalyse the formation of knobs on the erythrocyte plasma membrane and an overall increase in erythrocyte rigidity, presumably by modulating the endogenous erythrocyte cytoskeleton. In uninfected erythrocytes, Band 4.1 (4.1R) plays a key role in regulating erythrocyte shape by interacting with multiple proteins through the three lobes of its cloverleaf-shaped N-terminal domain. In *P. falciparum-*infected erythrocytes, the C-lobe of 4.1R interacts with the *P. falciparum* protein mature parasite-infected erythrocyte surface antigen (MESA), but it is not currently known whether other *P. falciparum* proteins bind to other lobes of the 4.1R N-terminal domain.

**Methods:**

In order to identify novel 4.1R interacting proteins, a yeast two-hybrid screen was performed with a fragment of 4.1R containing both the N- and α-lobes. Positive interactions were confirmed and investigated using site-directed mutagenesis, and antibodies were raised against the interacting partner to characterise it’s expression and distribution in *P. falciparum* infected erythrocytes.

**Results:**

Yeast two-hybrid screening identified a positive interaction between the 4.1R N- and α-lobes and PF3D7_0402000. PF3D7_0402000 is a member of a large family of exported proteins that share a domain of unknown function, the PHIST domain. Domain mapping and site-directed mutagenesis established that it is the PHIST domain of PF3D7_0402000 that interacts with 4.1R. Native PF3D7_0402000 is localized at the parasitophorous vacuole membrane (PVM), and colocalizes with a subpopulation of 4.1R.

**Discussion:**

The function of the majority of *P. falciparum* exported proteins, including most members of the PHIST family, is unknown, and in only a handful of cases has a direct interaction between *P. falciparum*-exported proteins and components of the erythrocyte cytoskeleton been established. The interaction between 4.1R and PF3D7_0402000, and localization of PF3D7_0402000 with a sub-population of 4.1R at the PVM could indicate a role in modulating PVM structure. Further investigation into the mechanisms for 4.1R recruitment is needed.

**Conclusion:**

PF3D7_0402000 was identified as a new binding partner for the major erythrocyte cytoskeletal protein, 4.1R. This interaction is consistent with a growing body of literature that suggests the PHIST family members function by interacting directly with erythrocyte proteins.

## Background

Intraerythrocytic *Plasmodium falciparum* parasites extensively remodel their host cells. As the parasite matures the erythrocyte becomes more rigid, loses its biconcave shape for a more spherocytic one, and small electron dense protrusions, known as knobs, form on the surface [1]. These modifications enable the infected erythrocyte to adhere to and block the microvasculature and microcapillaries, which in turn contributes to specific complications such as cerebral and placental malaria [2,3]. *Plasmodium falciparum* achieves these massive changes in erythrocyte structure by exporting proteins into the erythrocyte cytosol, some of which interact with components of the host cytoskeleton and plasma membrane [3]. Only a few of these exported proteins have been studied in detail, such as knob-associated histidine rich protein (KAHRP), an essential structural component of knobs that secure the major cytoadherence ligand *P. falciparum* erythrocyte membrane protein 1 (PfEMP1) to the erythrocyte cytoskeleton through interactions with spectrin and actin [4-6], and mature parasite infected erythrocyte surface antigen (MESA), which binds to the erythrocyte cytoskeleton associated protein Band 4.1, also known as 4.1R [7,8].

For the majority of the more than 400 *P. falciparum* proteins that contain an export motif, referred to as the *Plasmodium* Export Element (PEXEL), little is know about their function [3,9,10]. This includes a group of 71 exported proteins that share a conserved domain of four alpha helices in tandem, termed the *Plasmodium* helical interspersed subtelomeric (PHIST) domain. PHIST domain-containing proteins can subdivided into three groups, PHISTa, PHISTb, and PHISTc, according to the position of one or two conserved tryptophans in the PHIST domain [11]. Several genes encoding PHIST proteins have been knocked out as part of a large-scale screen for exported protein function, and some of these knockouts showed a decrease in erythrocyte rigidity, implying that at least some PHIST proteins may play a role in modulating the erythrocyte cytoskeleton [12]. One PHISTb family member, ring infected surface antigen (RESA), is known to interact directly with the erythrocyte cytoskeleton by binding to spectrin tetramers, resulting in increased resistance to shear stress and thermal damage [13], while another binds to the C-terminal tail of PfEMP1 [14] but in the majority of cases no binding partner has been identified.

4.1R is involved in maintaining the biconcave shape, elasticity, and mechanical stability of human erythrocytes, and defects in 4.1R are one cause of hereditary erythrocyte elliptocytosis [15,16]. 4.1R is a member of a family of proteins that contain an N-terminal FERM domain, which regulates local cell shape by acting as a coordinator for protein-protein interactions [17]. Other FERM domain containing proteins, such as Ezrin, Radixin and Moesin, cross-link actin to the cytoplasmic tails of CD43, CD44, and ICAM-1 and have been implicated in regulation of cellular protrusions such as microvilli [18,19]. The 4.1R FERM domain interacts with erythrocyte membrane proteins Band 3, Glycophorin C and p55 through its N-, α- and C-lobes, respectively, linking them to the actin-spectrin network, which is bound by the 4.1R C-terminal domain [20-22].

Given the critical regulatory role that the 4.1R FERM domain plays in uninfected erythrocytes, it is not surprising that *P. falciparum* proteins also bind to it, and the C-lobe of the 4.1R FERM domain is recognized by MESA [23,24]. To test the hypothesis that the other lobes of the 4.1R FERM domain are also bound by exported *P. falciparum* proteins, a yeast two-hybrid screen of a *P. falciparum* cDNA library was performed using the N- and α- lobes of the 4.1R FERM domain as bait. This fragment of 4.1R interacted with PF3D7_0402000, an uncharacterized member of the PHISTa family. The 4.1R-PF3D7_0402000 interaction was mapped to the single amino acid level using the yeast two-hybrid system and visualized by immunofluorescence in *P. falciparum-*infected erythrocytes.

## Methods

### *Plasmodium falciparum* culture

*Plasmodium falciparum* 3D7 strain parasites were cultured in O + human erythrocytes and serum (purchased from Interstate Blood Bank) as described previously [25] and synchronized by application of 5% sorbitol during the ring stages. Use of human erythrocytes and serum for culture of *P. falciparum* was approved by University of Alabama at Birmingham Institutional Review Board.

### Yeast two-hybrid screen

The Matchmaker GAL4 Two-Hybrid System 3 (Clontech) was used for yeast two-hybrid screens and analysis. A *P. falciparum* cDNA library containing activation domain fusions with fragments of *P. falciparum* proteins was kindly provided by Dr Lawrence Bergman (Drexel University). The 4.1R bait plasmid was made by cloning a fragment of the FERM domain of 4.1R (forward primer 5′-GGAATTCATGCACTGCAAGGTTTCTTTGTTGG-3′, reverse primer 5′- CGGGATCCTTAATGAAGATCAACTCCATAC-3′) into pGBKT7. The bait plasmid was transformed into yeast strain AH109, which was subsequently transformed with the *P. falciparum* cDNA-activation domain fusion library. Screening was performed by plating double transformants onto low stringency plates (−Leu/-Trp/-His) then replica plating onto high stringency plates (−Leu/-Trp/-His/-Ade) after four days growth. Activation domain plasmids were then rescued from all colonies that grew at high stringency, and retransformed into the original bait strain, as well as a strain transformed with an empty pGBKT7 vector, to test for plasmid independence and auto-activation.

### Yeast two-hybrid interaction validation and mapping

In order to recapitulate the interaction observed in the yeast-two-hybrid screen, a fragment of *PF3D7_0402000* was cloned into pGADT7 (forward primer 5′- GGAATTCGGTAATAAGGAAGATAACCAGG-3′, reverse primer 5′- GGGGATCCTTAACCTCCTTGTTTTATATTTTGC-3′). Once the 4.1R-PF3D7_0402000 interaction was confirmed, a series of deletion and point mutation constructs of PF3D7_0402000 were made. The positions of the deletions and mutations are described in the text, and were made using PCR primers as follows: ΔND1 construct (forward primer 5′-GGAATTCGAAAAGATATGGAATCATGCCG-3′, reverse primer 5′- GGGGATCCTTAACCTCCTTGTTTTATATTTTGC-3′), ΔCD1 construct (forward primer 5′- GGAATTCGGTAATAAGGAAGATAACCAGG-3′, reverse primer 5′-GTGGATCCTTAAGTATAATCATTTTCATGAAC-3′), ΔND3 (forward primer 5′-GGAATTCAAAGAACAAATGAAAAATTTAG-3′, reverse primer 5′-GGGGATCCTTAACCTCCTTGTTTTATATTTTGC-3), PHIST (forward primer 5′- GGGAATTCATGTTAAGTCAGGACTATAATGAT-3′ reverse primer 5′-GGGGATCCTTAGTCTATAGGAATTTCCGTAG-3′). Subfragments of the PF3D7_0402000 PHIST domain were created as follows: PHISTΔC1 (forward primer 5′-GGGAATTCATGCCCCGAAACGATATGGAAAAGATATGG-3′, reverse primer 5′-GGGGATCCTTAGTCTATAGGAATTTCCGTAG-3′), PHISTΔC4 (forward primer 5′- GGGAATTCATGTTAAGTCAGGACTATAATGAT-3′, reverse primer 5′-GGGGATCCTTAAGGGTCTTTATCAAGTAGTTC-3′), PHISTΔC1C4 (forward primer 5′-GGGAATTCATGCCCCGAAACGATATGGAAAAGATATGG-3′, reverse primer 5′ -GGGGATCCTTAAGGGTCTTTATCAAGTAGTTC-3′). Mutation of the conserved W residue within the PF3D7_0402000 PHIST domain was completed by PCR sewing (forward primer 5′GGGAATTCATGCAGTTAACAAAAGAAGAATTATATG-3′, and reverse primer 5′-GGGGATCCTTAGTCTATAGGAATTTCCGTAG-3′, internal forward primer 5′- AACGATATGGAAAAGATAGCTAATCATGCCGTTAAAACA-3′ and internal reverse primer 5′- TGTTTTAACGGCATGATTAGCTATCTTTTCCATATCGTT-3′). The PHIST domain from another PHIST family member was also cloned into pGADT7 as a control, using primers as follows: PF3D7_1253100 (forward primer 5′- GGGAATTCATGTTAAGTCAGGACTATAATGAT-3′, reverse primer 5′-GGGGATCCTTATTTTAATGAAAGAGTGTCC-3′).

All PF3D7_0402000 and PF3D7_1253100 constructs were cloned into pGADT7 and tested for interaction with the 4.1R bait plasmid in yeast strain AH109 at both medium (−Leu/-Trp/-His plates) and high stringency (−Leu/-Trp/-His/-Ade). Critical interactions were quantified by transforming the 4.1R bait and PF3D7_0402000 interacting plasmid into yeast strain Y187 (Clontech) and measuring β-galactosidase activity using the Pierce yeast β-galactosidase assay kit as directed by manufacturer instructions. All β-galactosidase assays were performed in triplicate.

### Expression of 4.1R and PF3D7_0402000 for production of polyclonal antisera

A fragment of human 4.1R was cloned from cDNA into the expression vector pRSETB (forward primer 5′-GAAGATCTATGCACTGCAAGGTTTCTTTGTTGG-3′, reverse 5′-CGGGATCCTTAATGAAGATCAACTCCATAC-3′) and a fragment of PF3D7_0402000 was cloned into pRSETA (forward 5′-CGGGATCCGGATAAATATGAAGGATATGCTGC-3′, reverse 5′-AACTGCAGTTAACCTCCTTGTTTTATATTTTGC-3′). Both plasmids were transformed into *Escherichia coli* BL21-DE3 pLysS. Recombinant proteins were expressed as hexa-his fusions and purified using either a Hi-Trap Chelating HP column (Amersham Biosciences) for the 4.1R fragment, or Nickel-NTA affinity agarose beads (Qiagen) for the PF3D7_0402000 fragment. The purified recombinant protein was then used to immunize both rabbits and rats (Cocalico Biologicals, Inc). PF3D7_0402000 rabbit antisera was affinity purified by coupling the purified hexa-his fusion protein to Hi-Trap NHS-activated beads (Amersham Biosciences), and passing the rabbit antisera over a column containing the NHS-activated beads. After extensive washing with 1X PBS, affinity-purified antibodies were eluted from the column with low pH buffer (0.1 M glycine, 0.5 M NaCl, pH 2.5–2.8) directly into a neutralization buffer (0.1 M Tris, pH 8.8) and dialysed for 24 hours against 1× PBS.

### SDS PAGE and Western blots

Parasite SDS lysates were made by resuspending *P. falciparum*-infected erythrocytes (1–3 × 10^8^ parasites/ml) in a 1× SDS sample buffer with 5% β-mercaptoethanol. Triton X100 extracts were made by resuspending *P. falciparum* infected erythrocytes (3 × 10^8^ parasites/ml) in an equal volume of 1% Triton X100 in PBS. After centrifugation, the supernatant was saved as the Triton soluble fraction and the pellet was washed three times in 1% Triton X100 in PBS. The pellet was resuspended in a 1× SDS sample buffer with 5% β-mercaptoethanol and labelled as the Triton X100 insoluble fraction. SDS lysates or Triton X100 extracts were loaded onto and run on 12% polyacrylamide gels and then transferred to nitrocellulose membranes. Membranes were probed with primary antibodies as described, followed by HRP-conjugated secondary sera (anti-rabbit or anti-rat), and visualized using enhanced chemoluminescence (ECL, Amersham Biosciences).

### Immunofluorescence

Air-dried thin smears of *P. falciparum* were either directly probed with anti-4.1R, anti-PF3D7_0402000, and/or PfSERA5, or were fixed with an ice-cold 50% acetone/50% methanol solution and washed with 1× PBS before probing with the appropriate primary antisera. Secondary antisera (Molecular Probes) labeled with a fluorophore were used for visualization on an Olympus BX60 fluorescence microscope equipped with a Diagnostic Instruments Spot RT monochrome camera. All images were merged with Adobe Photoshop 5.0. Anti-PfSERA5 [26] was a kind gift from Dr Brendan Crabb.

## Results

### The 4.1R FERM domain interacts with PF3D7_0402000 in a yeast two-hybrid screen

A fragment of the 4.1R FERM domain containing the N- and α-lobes was used as the bait in a yeast two-hybrid screen of a *P. falciparum* cDNA library (a kind gift from Dr Lawrence Bergman, Drexel University). Screening was performed in the yeast strain AH109 and transformants were originally plated at low stringency (−Leu/-Trp/-His), then transferred to high stringency plates (−Leu/-Trp/-His/-Ade) after four days of growth. More than 1 × 10^6^ colonies were screened and only nine grew on high stringency -Leu/-Trp/-His/-Ade plates. The plasmids from all nine positive colonies were isolated and retransformed with either the original 4.1R bait plasmid or an empty binding domain vector, pGBKT7, to test for plasmid independence and auto-activation. Two of the nine plasmids isolated from the screen did not recapitulate the growth on –Leu/-Trp/-His/-Ade plates and another three recapitulated the phenotype with the empty pGBKT7 vector, suggesting they were causing auto-activation.

**Figure 1:**
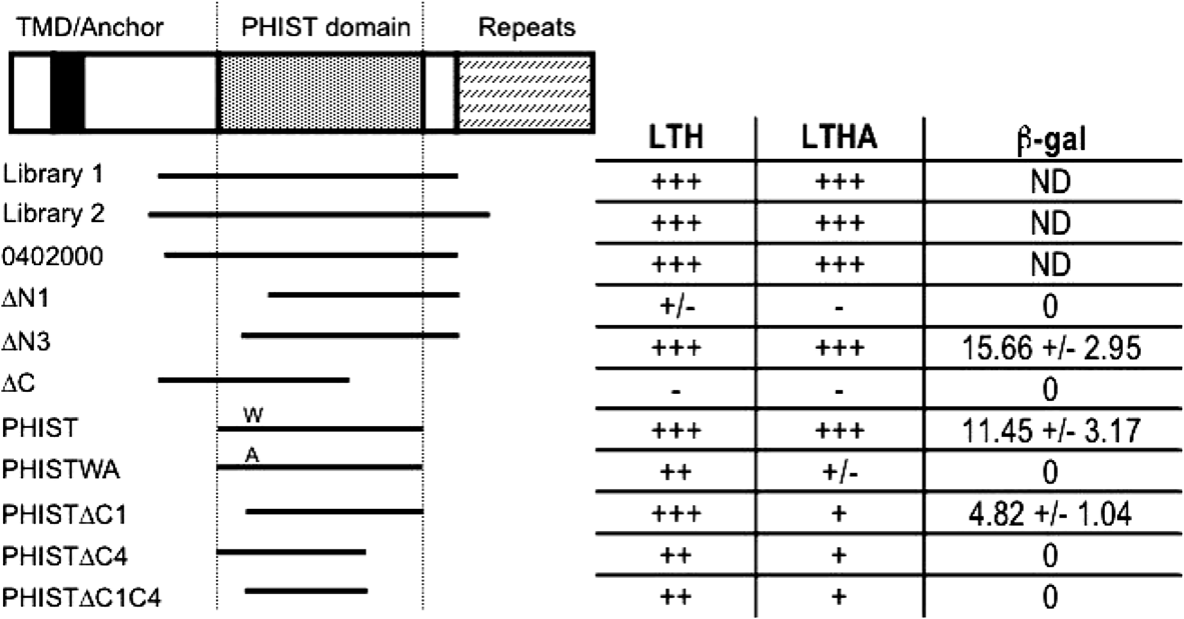
**The PF3D7**_**0402000 PHIST domain interacts with 4.1R.** Cartoon of PF3D7_0402000 showing the predicted transmembrane domain (TMD) or signal anchor, the PHISTa domain, and the C-terminal repeats. The regions of PF3D7_0402000 that were present in the rescued *P. falciparum* cDNA library plasmids (Library 1 and 2), or subsequently amplified and cloned into the yeast two-hybrid vector pGADT7 are indicated below the cartoon. The ability of each construct to interact with the 4.1R bait was scored at low (LTH: growth on –Leu/-Trp/-His plates) and high stringency (LTHA: growth on –Leu-/Trp/-His/-Ade plates) in strain AH109. Selected positive constructs were quantitatively scored for β-galactosidase activity assays in strain Y187 (assays performed in triplicate; data presented is the average +/− standard deviation between replicates).

The remaining four plasmids all recapitulated the phenotype of growth on high stringency plates in combination with the 4.1R bait plasmid, but not with an empty binding domain vector. Sequencing these plasmids revealed that all four contained fragments of the same *P. falciparum* gene, PF3D7_0402000. Three of the four plasmids contained identical inserts (Library 1; Figure 1), suggesting the same library clone was isolated independently three times, while the final plasmid contained a larger fragment of PF3D7_0402000 that overlapped in part with the other three plasmids (Library 2; Figure 1). To confirm the interaction a fragment of PF3D7_0402000 similar to library plasmid 1, but lacking six amino acids at the N-terminus, was amplified from genomic 3D7 DNA and cloned into pGADT7 (PF3D7_0402000; Figure 1). This fragment also supported growth on high stringency in combination with the 4.1R bait plasmid. The isolation of two overlapping fragments of PF3D7_0402000 in a yeast two-hybrid screen and the recapitulation of the interaction with an independent fragment of PF3D7_0402000 is strong evidence that the product of this gene interacts with the FERM domain of 4.1R.

### PF3D7_0402000 interacts with 4.1R through its PHIST domain

PF3D7_0402000 encodes a 50kDa protein that is predicted to contain a hydrophobic TMD/anchor sequence, a *P. falciparum* export motif, and a PHIST domain (Figure 1). PHIST domains define a family of 71 *P. falciparum*-exported proteins and consist of four tandemly arrayed alpha helices. The presence and location of two conserved tryptophan residues within the PHIST domain has been used to subdivide the PHIST family into three subfamilies. PF3D7_0402000 is a member of the PHISTa sub-family, which contains 23 members and unlike other PHIST subfamilies appears to be *P. falciparum* specific [11]. Following the PF3D7_0402000 PHIST domain is a series of repeated low-complexity regions at the C-terminal end, which is absent from both interacting plasmids isolated from the library.

In order to define the minimal domain of PF3D7_0402000 that interacts with 4.1R, a series of smaller fragments of PF3D7_0402000 were cloned into pGADT7 and tested for interaction with the 4.1R bait vector on high stringency plates. When a portion of the C-terminal end of the library plasmid 1 was deleted, (ΔC), no growth was observed on Leu/-Trp/-His/-Ade– plates, suggesting this region is necessary for the PF3D7_0402000/4.1R interaction (amino acids 249–325). Deletion of an N-terminal fragment from the same plasmid, (ΔN1), also inhibited interaction with 4.1R, whereas a smaller N-terminal deletion, (ΔN3), allowed growth on both –Leu/-Trp/-His/and –Leu/-Trp/-His/-Ade plates, suggesting that this region is dispensable for interaction with 4.1R (Figure 1).

These preliminary deletions suggested that it may be the PHIST domain of PF3D7_0402000 that interacts with 4.1R, as the PHIST domain is largely preserved in all the deletion constructs that grew on high stringency plates. To test this hypothesis, the entire PHIST domain of PF3D7_0402000 (amino acids 160–300) was cloned into pGADT7 and tested for interaction with the 4.1R-bait plasmid. The PF3D7_0402000 PHIST domain alone was sufficient for interaction with 4.1R, as growth was observed on both –Leu/-Trp/-His and –Leu/-Trp/-His/-Ade plates (PF3D7_0402000-PHIST; Figure 1). The interaction between the PF3D7_0402000 PHIST domain and 4.1R is specific, as the PHIST domain from another PHISTa family member, PF3D7_1253100, was not able to interact with the 4.1R bait plasmid (not shown).

### The conserved tryptophan in the PF3D7_0402000 PHIST domain is necessary for interaction with 4.1R

**Figure 2:**
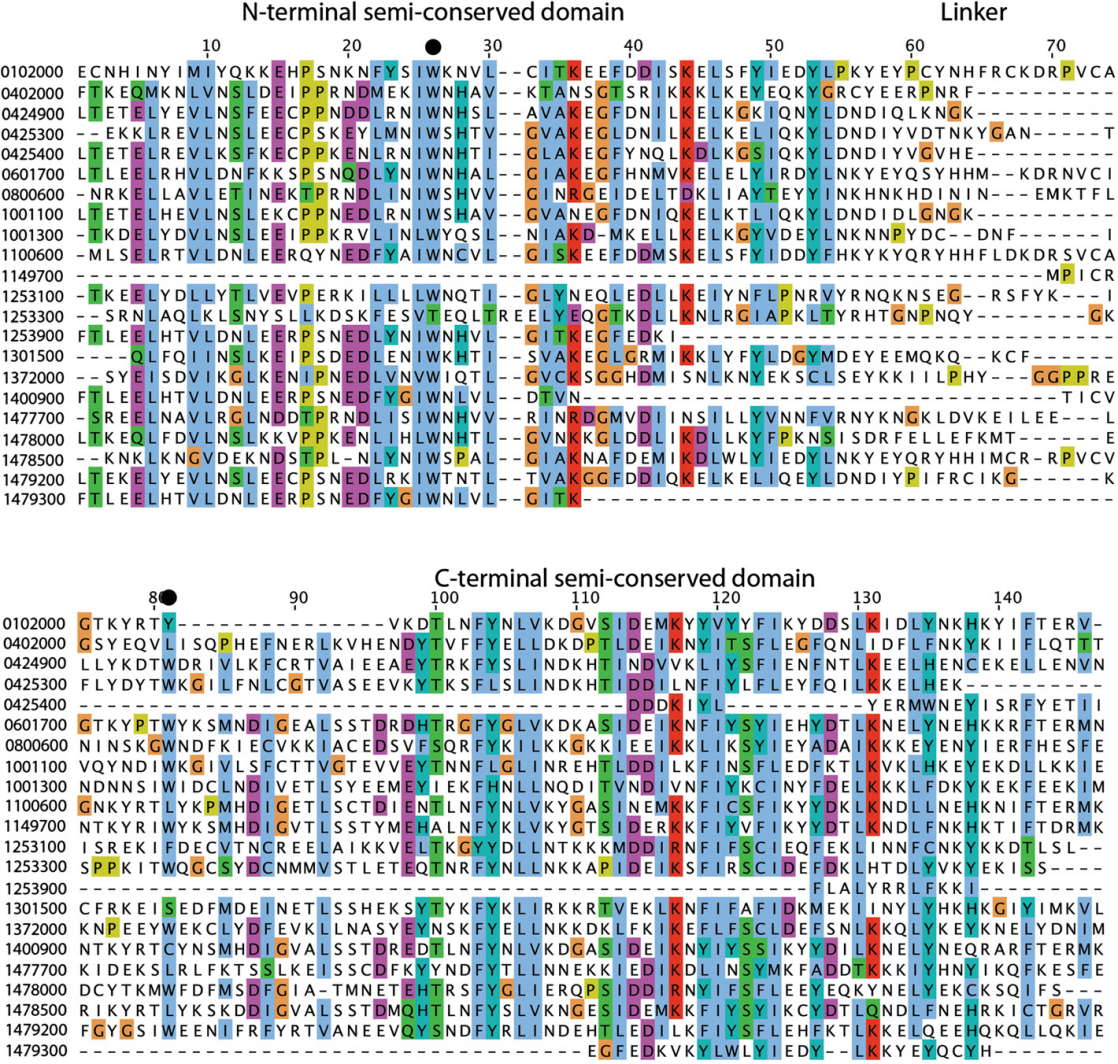
**Alignment of PHIST domains from PHISTa family members.** The PHISTa domains of all *P. falciparum* PHISTa family members were aligned using MUSCLE [27] and edited using Jalview [28]. Residues are coloured according to the standard CLUSTAL colour scheme: blue indicates a majority of hydrophobic amino acids at a given residue, red indicates a majority of basic amino acids, magenta indicates a majority of acidic amino acids, green indicates a majority of N, S, T or Q, and G and P are coloured regardless of conservation. The two tryptophan residues that are conserved in most PHIST domains are marked by closed circles.

The PHIST domain is defined largely by conserved structural predictions (four alpha helices in tandem of consistent sizes) rather than primary sequence conservation [11]. Alignment of the amino acid sequences of PHISTa domains reinforces this point, and suggests that the consensus PHISTa domain consists of two semi-conserved domains linked by a poorly conserved spacer fragment that in several proteins has one or more helix-breaking proline residues (see Figure 2). Some of the smaller PHISTa domains contain only the N-terminal semi-conserved domain (PF3D7_1253900, for example), while others contain only the C-terminal semi-conserved domain (PF3D7_1149700, for example). As previously reported, among the most conserved residues in the PHISTa domains are two tryptophans, one in each semi-conserved domain. Most PHISTa family members contain both tryptophan residues (see Figure 2, marked with closed circles at residues 26 and 81), but PF3D7_0402000 contains only the first one. To test the hypothesis that this conserved tryptophan residue is important for the interaction of PF3D7_0402000 with 4.1R, it was mutated to alanine in the PF3D7_0402000 PHIST domain construct. This point mutant allowed for growth on –Leu/-Trp/-His but only slow growth on –Leu/-Trp/-His/-Ade PHISTWA; (Figure 1). To quantify the interaction, β-galactosidase activity was measured in the strain Y187, which is a more stringent test (see Methods). No activity was detected when PHISTWA and the 4.1R bait plasmid were co-expressed in Y187, indicating that the conserved tryptophan is needed for an efficient interaction between PF3D7_0402000 and 4.1R.

To test whether all four PHIST helices are necessary for PF3D7_0402000 binding to 4.1R, the first and fourth helices were deleted, both alone and together, from the PHIST binding domain construct. Deletion of either coil 1 or coil 4 of the PHIST domain individually (PHISTΔC1 and PHISTΔC4) or both helices together (PHISTΔC1C4), did not completely eliminate the interaction with 4.1R as all three constructs allowed growth on both –Leu/-Trp/-His and –Leu/-Trp/-His/-Ade plates (Figure 1). However, when β-galactosidase assays were performed it became apparent that the full PHIST domain was needed for maximal interaction with 4.1R. Deletion of both coils 1 and 4 together, or coil 4 alone, completely eliminated β-galactosidase activity, while deletion of coil 1 had only minimal β-galactosidase activity (Figure 1). Taken together, the results suggest that neither coil 1 nor coil 4 are absolutely required for interaction with 4.1R, but the full PHIST domain is required for maximal interaction as determined by β-galactosidase assays.

### PF3D7_0402000 is expressed at the parasitophorous vacuole membrane

**Figure 3:**
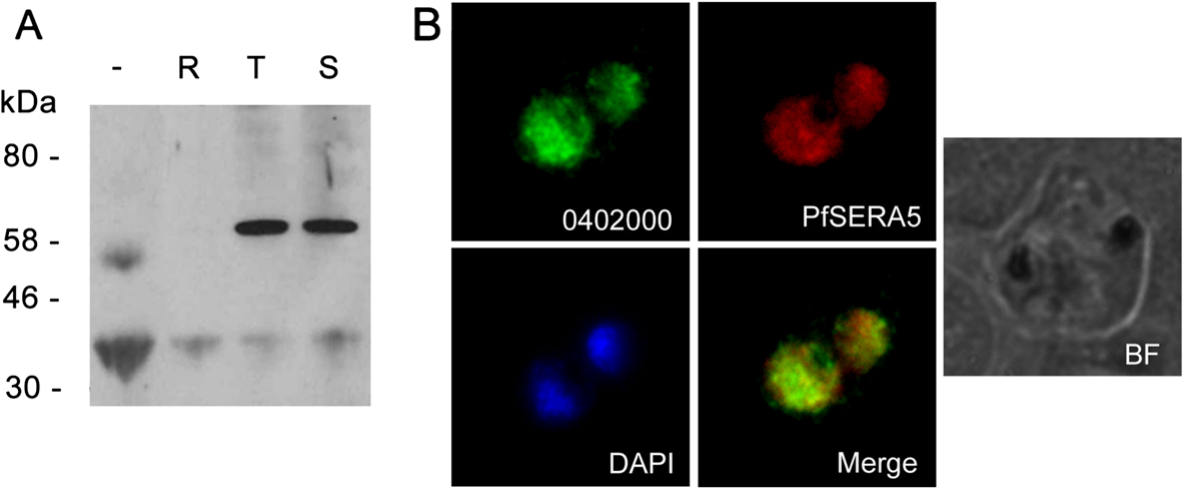
**PF3D7_0402000 localizes to the parasitophorous vacuole membrane. (A)** Total SDS extracts from uninfected erythrocytes (−), or erythrocytes infected with ring (R), trophozoite (T) or schizont (S) stage 3D7 *P. falciparum* parasites were probed with rat anti-PF3D7_0402000 polyclonal antisera. PF3D7_0402000 is expressed in trophozoite and schizont stage parasites, consistent with microarray data. **(B)** Fixed smears probed with rat anti-PF3D7_0402000 (0402000) and anti-PfSERA5 (SERA5) to test for co-localization (Merge). Parasite nuclei were identified with the nuclear stain DAPI, and infected erythrocytes imaged in brightfield (BF).

To determine the localization of endogenous PF3D7_0402000 in infected erythrocytes, both rabbit and rat polyclonal antibodies were raised against a purified hexa histidine-tagged fragment of PF3D7_0402000 that spans from the end of the putative transmembrane, representing amino acids 51–325 of the predicted protein sequence. Western blots of *P. falciparum* extracts probed with the resulting rat anti-PF3D7_0402000 antibody predominantly identified a single band at approximately 60 kDa, larger than the predicted size of 50 kDa, or of 41kDa for a PEXEL processed form of PF3D7_0402000 (Figure 3A). One possible explanation for the unexpected mobility is that the antibodies are primarily recognizing a form of PF3D7_0402000 where PEXEL cleavage has not occurred. This would seem to be unlikely, however, because the PEXEL cleavage site is at amino acid residue 72, meaning that only 15 amino acids of sequence prior to the predicted cleavage site are present in the recombinant protein used to generate the polyclonal antibodies, compared to 259 amino acids of the predicted mature protein. However, the PF3D7_0402000 protein sequence does contain an extended unstructured region, and such regions are known to cause aberrant mobility of *P. falciparum* proteins in SDS-PAGE.

Expression of PF3D7_0402000 was detected in trophozoite and schizont extracts, but not early ring stage extracts or in uninfected erythrocytes (Figure 3A), consistent with published *P. falciparum* microarray data, which show peak expression of PF3D7_0402000 in 3D7 at approximately 24 hours [29,30]. To determine the intracellular location of PF3D7_0402000, affinity-purified anti-PF3D7_0402000 antibodies were used in immunofluorescence experiments. Air-dried smears of 3D7 parasites were co-stained with specific antibodies against PF3D7_0402000 and PfSERA5. A significant portion of PF3D7_0402000 colocalized with PfSERA5 (Figure 3B), suggesting that PF3D7_0402000 is a PVM protein.

### A sub-population of 4.1R colocalizes with PF3D7_0402000

**Figure 4:**
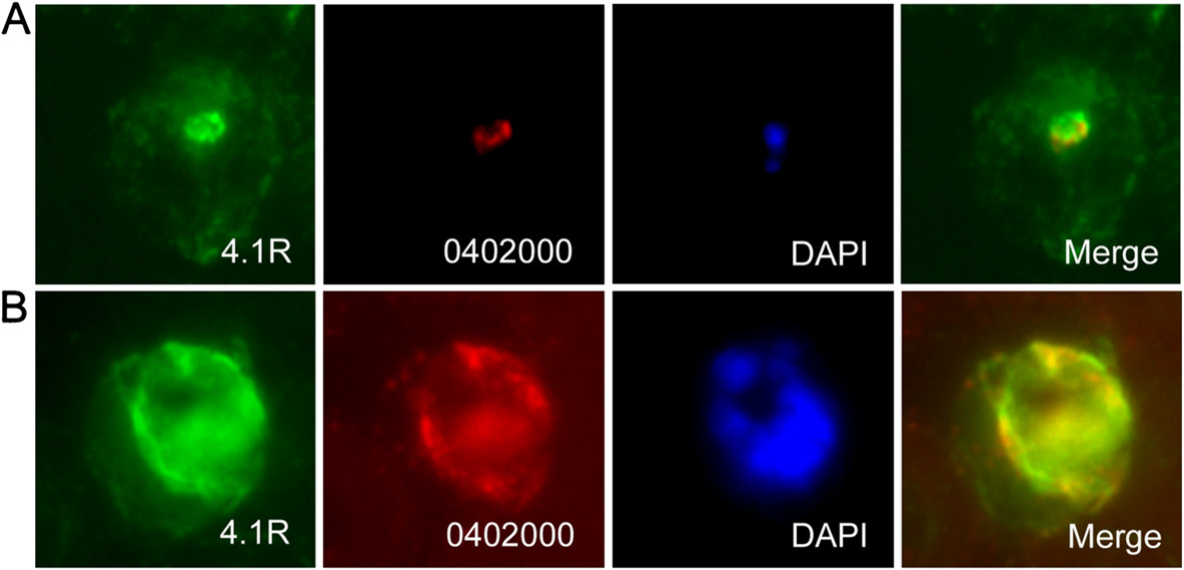
**PF3D7_0402000 colocalizes with a sub-population of 4.1R.** Immunofluorescence microscopy of air-dried smears of 3D7 *P. falciparum*-infected erythrocytes probed with rabbit anti-4.1R (green), rat anti-PF3D7_0402000 (red) and the nuclear dye (DAPI). PF3D7_0402000 and 4.1R colocalize in early **(A)** and late **(B)** trophozoite stage parasites.

**Figure 5:**
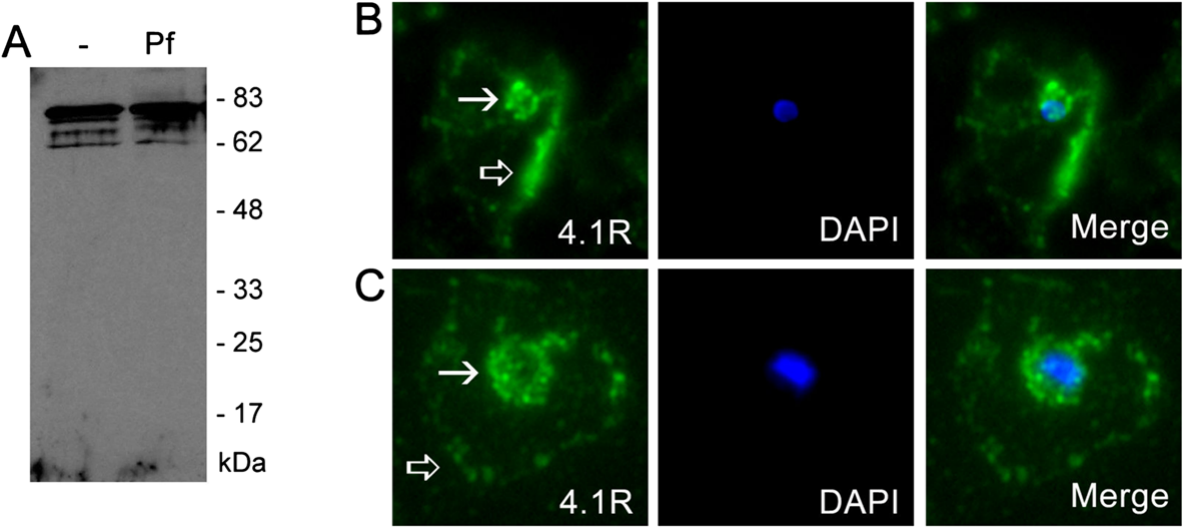
**A subpopulation of 4.1R is found at the PVM in *Plasmodium falciparum-*infected erythrocytes. (A)** Rabbit polyclonal antibodies raised against the FERM domain of 4.1R recognize a major band at the size of full-length 4.1R (80kDa) in both uninfected (−) and *P. falciparum*-infected (Pf) erythrocytes. Additional bands are either proteolytic cleavage products or alternatively spliced products of 4.1R as the same bands were recognized in both uninfected and *P. falciparum-*infected erythrocytes. **(B, C)** Immunofluorescence microscopy of air-dried smears of 3D7 *P. falciparum*-infected erythrocytes probed with rabbit anti-4.1R (green). 4.1R consistently localizes near the erythrocyte plasma membrane (black arrows), but in early (**B**) and late (**C**) trophozoite-stage parasites, a subpopulation of 4.1R is found in close apposition to the parasite, as detected by DAPI staining (blue).

In order to confirm that 4.1R and PF3D7_0402000 interact *in vivo*, co-immunoprecipitation experiments were attempted but were thwarted by the fact that 4.1R is detergent insoluble in both uninfected and infected erythrocytes (data not shown), making the recovery of detectable amounts of 4.1R from detergent extracts by immunoprecipitation impossible. However, co-immunofluorescence with antibodies against PF3D7_0402000 and 4.1R show that a subpopulation of 4.1R clearly colocalizes with PF3D7_0402000 at the PVM (Figure 4A, B). The presence of 4.1R at the PVM is not due to cross-reaction with *P. falciparum* proteins, as the anti-4.1R antisera recognizes exactly the same fragments in SDS extracts from uninfected (−) and *P. falciparum* infected (Pf) erythrocytes, with a major band at 80 kDa, the size of mature 4.1R (Figure 5A). 4.1R is by no means completely relocalized to the PVM in infected erythrocytes, as a significant proportion of 4.1R staining remains at the erythrocyte periphery (black arrow, Figure 5B, C) consistent with the known interactions of 4.1R with the erythrocyte plasma membrane proteins glycophorin C and Band 3, and the *P. falciparum* protein MESA. However, some 4.1R is clearly found at the PVM, consistent with an interaction of a subpopulation of 4.1R with PF3D7_0402000.

## Discussion

During the intraerythrocytic life cycle of *P. falciparum*, the parasite is predicted to export between 400–600 proteins into the erythrocyte cytosol, based on the presence of a motif (termed PEXEL) that is required for the export of many known exported proteins [9,10]. The function of many of these exported proteins is unknown. PfEMP1, KAHRP, and MESA are all *P. falciparum*-exported proteins that interact with the erythrocyte cytoskeleton and play a role in changing the structure of the erythrocyte membrane and cytoskeleton. The PHIST proteins, originally identified through searching the *P. falciparum* genome for PEXEL-containing proteins [11], may play a similar role, as knockouts of several PHIST family members show a decrease in erythrocyte rigidity [12]. In this study, one member of the PHIST family, PF3D7_0402000, was shown to interact with the FERM domain of 4.1R in a yeast two-hybrid screen. Formal confirmation of the interaction by co-immunoprecipitation was not possible due to the insolubility of 4.1R, but it should be pointed out that a complex between MESA and 4.1R has not been co-immunoprecipitated from infected erythrocytes either, presumably for the same reason. However PF3D7_0402000 colocalizes with a subpopulation of 4.1R, supporting an interaction *in vivo*.

How could only a subpopulation of 4.1R be recruited to the PVM? Interestingly, increased phosphorylation of 4.1R has been observed in *P. falciparum*-infected erythrocytes, [31,32]. Phosphorylation of 4.1R has been shown to weaken its affinity for glycophorin C, actin, and spectrin and takes place sometime between early ring and early trophozoite stages [33]. It is possible that phosphorylation, or some other post-translational modification event that occurs in *P. falciparum-*infected erythrocytes, divides 4.1R into two distinct populations: one that remains associated with erythrocyte cytoskeleton and one that dissociates from the erythrocyte cytoskeleton and is available to interact with PF3D7_0402000 at the PVM. This model is clearly hypothetical, but should be readily testable. Relocalization of erythrocyte cytoskeleton components to the PVM in *P. falciparum*-infected erythrocytes is not without precedent. Erythrocytes contain a class II myosin, which also appears to partially relocalize around the PVM (31). Moreover, it has been shown that 4.1R can bind to this class II myosin through its spectrin and actin-binding domain [34]. Thus, it is possible that relocalized 4.1R binds and interacts with relocalized myosin as well as PF3D7_0402000 at the PVM.

The PVM is a dynamic membrane, which expands as the parasite divides and increases in size inside the erythrocyte. Given the role of 4.1R in maintaining structure at the erythrocyte surface, the presence of both 4.1R and myosin at the PVM is clearly consistent with a role in maintaining the stability of the PVM during this expansion process. It is also possible that 4.1R at the PVM plays a role in protein transport. Both the tubovesicular network, which consists of threadlike projections of the PVM that extend into the erythrocyte cytosol, and the formation of Maurer’s clefts, which may bud from the PVM [35,36], are modifications of the PVM. Other FERM domain-containing proteins, such as ezrin, radixin and moeisin, promote cellular protrusions such as microvilli [18,19]. 4.1R could perform a similar function at the PVM and be involved directly in TVN and/or Maurer’s cleft formation and budding, perhaps by cross-linking the PVM with other structural components, whether host or parasite derived. These hypothetical roles for 4.1R at the PVM remain to be tested.

The PHIST domain and PHIST family proteins were originally identified through bioinformatic approaches based on conserved structural features [10]. The identification of an interaction between the PHIST domain of PF3D7_0402000 and 4.1R allowed direct testing of whether the bioinformatic parameters used to define the PHIST families are functionally relevant. While neither the first or fourth helix or the conserved tryptophan residue in the PF3D7_0402000 PHISTa domain were absolutely necessary for interaction with 4.1R, the full PHIST domain and the conserved tryptophan were all required for a highly stringent interaction as measured by β-galactosidase activity. The broad outlines of the PHIST domain described by bioinformatics do therefore appear to have functional relevance. Knockout of several PHIST family members shows a decrease in erythrocyte rigidity [11]. Given the extensive remodelling of the erythrocyte cytoskeleton that occurs after infection by the *P. falciparum* parasite, it would seem reasonable to hypothesize that the PHIST domain has evolved to bind directly with an array of endogenous erythrocyte proteins, including components of the cytoskeleton, with individual PHIST domain proteins recognizing specific, and probably overlapping, targets. The PHIST family may therefore represent a central front in the parasite-host interactions that drive the successful colonization of human erythrocytes by *P. falciparum* parasites.

## Competing interests

The authors declare that they have no competing interests.

## Authors’ contributions

LP, JR, DM, and MJ designed the experiments: LP, MJ, DM, and EK performed the experiments: LP, JR, DM, and MJ analysed the data: LP and JR wrote the paper. All authors read and approved the final manuscript.
